# NetProphet 3: a machine learning framework for transcription factor network mapping and multi-omics integration

**DOI:** 10.1093/bioinformatics/btad038

**Published:** 2023-01-24

**Authors:** Dhoha Abid, Michael R Brent

**Affiliations:** Center for Genome Sciences and Systems Biology, Washington University School of Medicine, St. Louis, MO 63110, USA; Department of Computer Science and Engineering, Washington University, St. Louis, MO 63130, USA; Center for Genome Sciences and Systems Biology, Washington University School of Medicine, St. Louis, MO 63110, USA; Department of Computer Science and Engineering, Washington University, St. Louis, MO 63130, USA; Department of Genetics, Washington University School of Medicine, St. Louis, MO 63110, USA

## Abstract

**Motivation:**

Many methods have been proposed for mapping the targets of transcription factors (TFs) from gene expression data. It is known that combining outputs from multiple methods can improve performance. To date, outputs have been combined by using either simplistic formulae, such as geometric mean, or carefully hand-tuned formulae that may not generalize well to new inputs. Finally, the evaluation of accuracy has been challenging due to the lack of genome-scale, ground-truth networks.

**Results:**

We developed NetProphet3, which combines scores from multiple analyses automatically, using a tree boosting algorithm trained on TF binding location data. We also developed three independent, genome-scale evaluation metrics. By these metrics, NetProphet3 is more accurate than other commonly used packages, including NetProphet 2.0, when gene expression data from direct TF perturbations are available. Furthermore, its integration mode can forge a consensus network from gene expression data and TF binding location data.

**Availability and implementation:**

All data and code are available at https://zenodo.org/record/7504131#.Y7Wu3i-B2x8.

**Supplementary information:**

Supplementary data are available at *Bioinformatics* online.

## 1 Introduction

A transcription factor (TF) network map shows which TFs regulate each gene in a genome. Nodes represent genes and the proteins they encode; edges connect TFs to their direct, functional targets. A gene is a direct, functional target of a TF if the TF binds to the gene’s regulatory DNA and thereby modulates its transcription rate. Edges indicate the potential of a TF to regulate a gene under some conditions, but do not specify those conditions. The requirement that edges be direct provides a molecular semantics to the network map and makes it useful for re-engineering regulatory networks to change cellular behaviors. Indirect edges, which do not reflect molecular interactions, cannot be directly modified by genetic engineering. In addition to providing a roadmap for engineering regulatory networks, accurate network maps provide essential reference information for a wide range of biological investigations.

Several experimental methods aim to measure where a TF binds, including ChIP-Chip (Harbison *et al.*, 2004), ChIP-Seq, ChIP-Exo (Perreault and Venters, 2016; Rhee and Pugh, 2011; Rossi *et al.*, 2018) and transposon calling cards (Mayhew and Mitra, 2016; Wang *et al.*, 2007, 2011, 2012). However, TFs can bind to a gene’s regulatory DNA without affecting its transcription rate, so even TF network maps built from accurate binding location data include many non-functional edges (Cusanovich *et al.*, 2014; Gitter *et al.*, 2009; Kang *et al.*, 2020; Lenstra and Holstege, 2012). ChIP-Chip and ChIP-Seq are also subject to a host of artifacts (Brent, 2016) and yield many binding events even for non-DNA-binding proteins, such as green fluorescent proteins (Teytelman *et al.*, 2013). The only comprehensive binding dataset for nearly all TFs in a eukaryotic organism is a ChIP-Chip dataset for baker’s yeast, *Saccharomyces cerevisiae* (Harbison *et al.*, 2004). Newer methods, including ChIP-Exo and Transposon Calling Cards, yield fewer non-functional binding events (Kang *et al.*, 2020), but data are currently available for only a few dozen yeast TFs.

A simple approach for predicting edges that are both direct and functional is to calculate the overlap between genes whose promoters are bound by a TF in ChIP-Chip experiments and those that respond to a direct perturbation to that TF. However, this overlap is typically very small (Gitter *et al.*, 2009; Kang *et al.*, 2020). A much better overlap can be achieved by processing perturbation response data through TF network mapping algorithms (Kang *et al.*, 2020). Most such algorithms build on the idea that correlation between the mRNA levels of a TF and those of a gene suggests that the TF may regulate the gene. In this approach, TF network maps are constructed by regressing target gene mRNA levels on TF mRNA levels (Bonneau *et al.*, 2006; Greenfield *et al.*, 2010; Huynh-Thu *et al.*, 2010; Huynh-Thu and Geurts, 2018; Jackson *et al.*, 2020; Madar *et al.*, 2009; Margolin *et al.*, 2006; Roy *et al.*, 2013; Siahpirani and Roy, 2017). However, there are drawbacks to this approach: mRNA levels of TFs may not be strongly correlated with those of their target genes. Furthermore, they can be correlated with genes the TF does not directly regulate.

For several years, an annual community competition called Dialogue for Reverse Engineering Assessment and Methods (DREAM) was held to evaluate gene expression-based network mapping algorithms. Some of the most successful approaches are LASSO regression (Gibbs *et al.*, 2022; Greenfield *et al.*, 2010; Haynes *et al.*, 2013; Kang *et al.*, 2017) and tree-based models (Gibbs *et al.*, 2022; Huynh-Thu and Geurts, 2018). These methods and the others described here produce a score for each possible (TF, target) pair, such that higher scores indicate a greater likelihood of being a true edge. We refer to these as evidence scores or weighted networks.

To predict a better TF network map, NetProphet (Haynes *et al.*, 2013) combined evidence scores from LASSO and differential expression (DE) analysis after direct perturbation of a TF. NetProphet 2.0 (NP2) (Kang *et al.*, 2017) combined the NetProphet output with two additional score matrices. One was derived by regressing gene expression levels on TF expression levels using Bayesian Additive Regression Trees (BART) (Chipman *et al.*, 2010). The other was derived by building a preliminary network, inferring binding motifs (PWMs) for each TF from the promoters of its predicted targets, and scoring the promoters of all genes with that PWM (Kang *et al.*, 2017). A limitation of NP2 is that it combined these scores using an elaborate, multi-stage procedure that was manually optimized on a particular dataset and may not be optimal for combining evidence scores from other sources.

Here, we introduce NetProphet3 (NP3), which learns to combine evidence scores from any sources by using a supervised machine learning framework. Each possible (TF, target) edge is an instance with features consisting of its evidence scores and binary labels based on whether there is evidence that the TF binds the target’s regulatory DNA. By default, NP3 uses the same evidence scores as NP2, but users are free to train on other input features. This makes it easy to incorporate evidence scores derived from multiple gene expression datasets (demonstrated here), scores from scanning known binding specificity models over regulatory DNA sequences, or scores from other network mapping algorithms. NP3 is an easy-to-use package that is distributed with a singularity container, so it can be run under any operating system without installing dependencies.

There are two main use cases for NP3. One is to predict the direct functional targets of a TF for which no binding data are available by using gene expression data. If there are enough TFs from the same organism that have binding data, they can be used to train NP3 to predict edges for TFs that do not have binding data. Otherwise, edges can be predicted by the provided model, which was trained on yeast data. The other use case involves TFs for which both perturbation-response data and binding data are available. Here, NP3 can be used to integrate evidence scores derived from gene expression or other data and labels derived from binding location data. To do this, NP3 is used to make predictions on the same instances it is trained on. The predicted scores are single numbers that integrate expression-based features and binding-based labels to form a consensus about the likelihood that a given (TF, target) pair is a direct, functional edge. This is a novel application of supervised machine learning in which the goal is not to generalize to unseen data but rather to form a consensus by integrating the features and labels of the training data. Since binding data are used as labels in training, their influence on the integrated score (i.e. the model’s prediction) can be increased by intentionally overfitting the model to the labels or reduced by under-fitting. We demonstrate that overfitting a model to binding data for one TF improves performance, according to metrics that are independent of the binding labels used in training.

We tested NP3 with two different expression datasets for yeast. In each dataset, most yeast TFs were perturbed, either by gene deletion or by transient overexpression in response to a chemical inducer. To evaluate performance, we then used three independent evaluation metrics: (i) the percentage of edges that are direct, estimated by the percentage of edges supported by binding data. (ii) The enrichment of gene ontology (GO) biological process terms among the predicted targets of each TF. (iii) The percentage of known protein–protein interactions (PPIs) among (TF, TF) pairs sharing many target genes. Improvement on all three metrics is a strong indicator of improved accuracy.

## 2 Materials and methods

### 2.1 NetProphet3

**Fig. 1.**
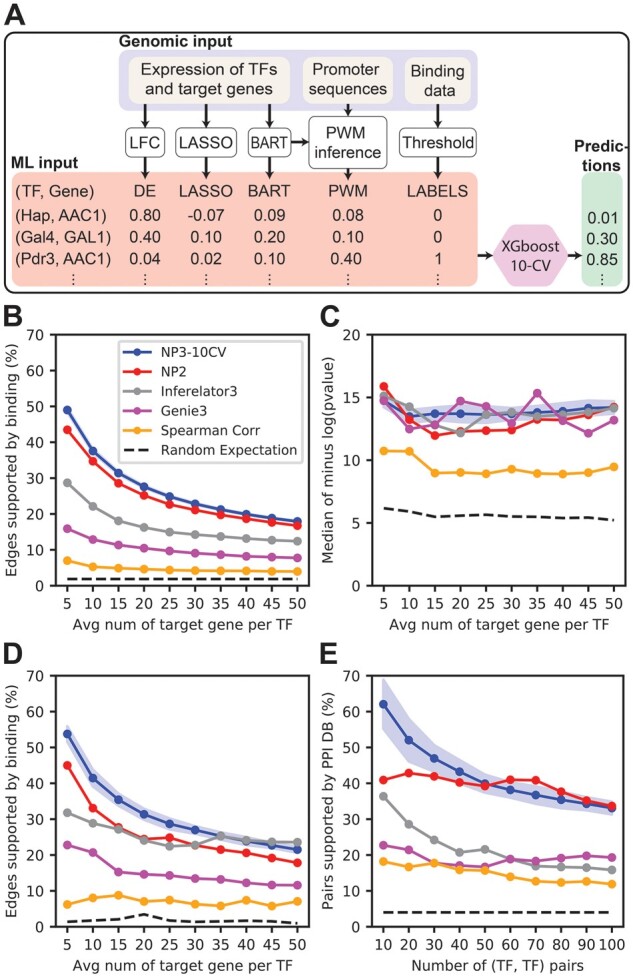
(**A**) NP3 combines evidence scores from DE, LASSO, BART and PWM by using XGBoost. Training instances are (TF, gene) edges. Features are the evidence scores, and labels are 1 if there is evidence of direct binding, 0 otherwise. NP3 is trained to predict the probability of an edge being supported by binding data. (**B**–**E**) Performance of NP3 and other network inference algorithms (average of TFKO and ZEV datasets). Blue: NP3 using 10-fold CV by TF; shaded area: 1 SD of 20 networks from different seeds for 10-CV. Shading is not shown for algorithms that do not require training. Red: NP2, fixed parameters; gray: Inferelator3 with DE prior; magenta: Genie3; orange: Spearman correlation; black dashed: random expectation. (**B**) Binding metric (defined in main text) calculated as a function of the number of top-scoring edges at different thresholds. NP3 outperforms all other network inference algorithms at all thresholds. Random expectation is the probability that a randomly selected (TF, gene) edge will be supported by binding data. (**C**) GO metric (defined in main text). NP3, NP2, Inferelator3, and Genie3 have similar performance. Random expectation is the GO metric for 20 NP3 networks in which TFs and genes were randomly permuted. (**D**) GO-directness metric (defined in main text). NP3 target predictions matching the TF’s most significant GO term have the highest percent binding support. Random expectation is the GO-directness of the GO results of the 20 random networks generated in (C). (**E**) PPI metric (defined in main text). Horizontal axis: number of top (TF, TF) pairs included. Vertical axis: percent of included (TF, TF) pairs supported by strong evidence of physical interaction. NP3 performs better than other algorithms for the top 40 pairs and about the same as NP2 on the top 50–100 pairs. Random expectation is the probability that a randomly selected (TF, TF) pair will be supported by the PPI STRING database.

NP3 combines four weighted networks: DE, LASSO, BART and PWM by using XGBoost (Fig. 1A). LASSO and BART scores are based on regressing each gene’s mRNA levels on those of all TFs. DE scores consist of the log_2_ fold-change (LFC) of gene expression profiles after a perturbation of a TF, except LFC between −0.38 and 0.38 were replaced by zeros (see Supplementary Material for details). PWM scores are computed by using a preliminary network (here derived from BART scores) to infer a binding motif from the promoter regions of the top predicted targets of each TF. The inferred motif for each TF is scanned across the promoters of all genes to create (TF, gene) scores. These four weighted networks form a set of four features that are trained with binary labels extracted from binding experiments: 1 when there is evidence of direct binding and 0 otherwise (see Section 2.4 below). XGBoost training depended on the use case. For predicting edges for TFs that do not have binding data (generalization mode), we trained it using 10-fold cross-validation (CV) on TFs. To construct a fold, we stratified TFs based on the number of their positively labeled targets, then selected 90% of TFs from each group at random for the training fold and the rest for the test fold. We tuned the hyperparameters of XGBoost using 5-fold CV within the training fold; the optimal parameters were used for training an XGBoost model with all the training data. For predicting targets of TFs that have binding data (integration mode), we made predictions on the same TF(s) that we trained on, using those predictions as the NP3 output score. For the cross-trained mode, we trained a model with all TFs from the TFKO dataset and tested that model on the ZEV dataset, and vice-versa. A TF was not allowed to regulate itself.

### 2.2 Inferelator3

Ideally, Inferelator3 v0.5.6 requires a binary ‘prior’ network of targets for each TF. It extends an existing network with additional targets, which makes it impossible to use the binary binding network as prior and carry out CV by TF—if the binding data are required as prior for each TF, it cannot be used to evaluate the network. Instead, we constructed a prior network from the DE data by assigning 1 when the absolute value of shrunken log_2_ fold-change (LFC) of gene expression is greater than zero (ZEV data) or when the absolute value of LFC of gene expression is >0.4 (TFKO data). This prior includes only TFs that were perturbed by overexpression (ZEV data) or knock-out (TFKO data). We also constructed another prior that included all TFs—TFs that were not perturbed were assigned zero priors (Supplementary Fig. S10A–D).

### 2.3 Gene expression profiles

We used two gene expression datasets for *S.cerevisiae* in which each TF was perturbed either by deletion (Kemmeren *et al.*, 2014) (TFKO) or overexpression (Hackett *et al.*, 2020) (ZEV). The TFKO dataset includes 1485 gene expression profiles of strains in which a single gene was deleted from the genome, of which 281 are TFs. The ZEV dataset contains gene expression profiles at various time points after transient induction of 167 TFs using estradiol (Supplementary Files S1 and S2).

### 2.4 Curated binding data

We downloaded a curated list of binding edges from an earlier study of NetProphet version 1 (Haynes *et al.*, 2013). This binary network includes 186 TFs and 29 ,946 (TF, target) edges without quantitative edge scores, most of them from ChIP-Chip experiments. It was originally downloaded from the regulatory edges with binding support in YEASTRACT (Abdulrehman *et al.*, 2011) but, since YEASTRACT was not versioned and has changed significantly since then, it is no longer possible to get a similar network from that site. Many (though not all) of the edges in this YEASTRACT network are derived from the ChIP-Chip data of (Harbison *et al.*, 2004). More recent methods, including Transposon Calling Cards (Mayhew and Mitra, 2016; Shively *et al.*, 2019; Wang *et al.*, 2008) and ChIP-Exo (Holland *et al.*, 2019) yield higher-quality data (Kang *et al.*, 2020). So, we curated calling cards data for 15 TFs and ChIP-Exo (Holland *et al.*, 2019) data for 25 TFs. Binary ChIP-Exo edges were provided in Holland *et al.* (2019)Supplementary Data. For Transposon Calling Cards, we selected top-ranked edges based on the minus log_10_*P*-values. We collected 2181 edges from Calling Cards and 10 132 from ChIP-Exo. For TFs that have binding experiments from multiple technologies, we replaced their labels in the binary network from Haynes *et al.* (2013), first, by a Calling Cards experiment, if there was one, and if not by a ChIP-Exo experiment, if there was one. Each TF has labels from one single experiment (Supplementary File S4). The final curated binding edges include 186 TFs and 31 996 edges that were used as positive labels to train NP3 and to calculate the percentage of binding support for evaluation metrics.

### 2.5 Binding evaluation metric

We calculated the percent of top-scoring (TF, target) edges that are supported by binding edges above different thresholds. Top-scoring edges are defined by sorting edges from highest to lowest by the absolute values of their scores. Thresholds are scaled to the number of target genes per TF. This is an average so different TFs have different numbers of target genes at each threshold (see Supplementary Fig. S5 for more details). The advantage of this method is that the number of edges at each threshold is proportional to the number of TFs in the network: a network with a greater number of TFs, such as those for mammalian organisms, will have a greater number of edges evaluated at each threshold. If the network has TFs that cannot be evaluated because no binding experiments are available for them, their edges are removed before thresholds and binding support are calculated. A higher percentage of binding support implies that the predicted network is enriched with direct edges.

### 2.6 GO and GO-directness evaluation metrics

For each TF’s targets, we did GO enrichment analysis using GO-Term-Finder v0.86 (Boyle *et al.*, 2004). In order to focus on terms associated with specific biological processes, rather than extremely generic terms like ‘biosynthetic process’, we excluded terms with more than 300 annotated genes. Then, each TF was assigned the GO term that had the highest minus log *P*-value. We reported the median of –log *P*-values for TFs that had –log *P* > 4, as the goal was to compare the specificity of the target set for the top GO term, rather than whether the TF had a GO term at all. The median was calculated among all TFs that had a significant *P*-value according to the filters described earlier. We did these analyses for each threshold as in the binding metric, but without excluding TFs that did not have binding data available. Next, in order to determine whether improvements on the GO evaluation metric came at the cost of including indirect edges, we calculated, for each TF, the percentages of binding support for edges that matched the GO term having the highest minus log *P*-value. For each threshold, we reported the average percentage of binding support across TFs. We call this evaluation metric GO-directness.

### 2.7 Protein–protein interaction evaluation metric

First, we calculated the Jaccard similarity between the sets of target genes of each (TF, TF) pair; the Jaccard similarity is 1 when the target sets are the same and 0 when none of the targets in the sets are shared. Next, we sorted (TF, TF) pairs by Jaccard similarity and calculated the percent of the top 100 pairs that is supported by physical interactions from the PPI STRING database (Szklarczyk *et al.*, 2021). The database has 2614 interactions predicted with a high degree of confidence (STRING score ≥ 0.7). A higher percentage of PPI support means that (TF, TF) pairs are likely to work as part of a known physical protein complex. We did these analyses for the (TF, target) edges at threshold 25 targets per TF.

## 3 Results

### 3.1 NetProphet3 is more accurate than other commonly used algorithms when TF perturbation-response data are available

We systematically combined evidence scores from DE, LASSO, BART and PWM using XGBoost (Chen and Guestrin, 2016), a machine learning algorithm, to predict one final network. Each training instance was a potential (TF, target) edge, features were evidence scores, and labels were 1 for edges supported by evidence of direct binding and 0 for the remainder (Fig. 1A). We obtained labels from curated TF binding location experiments including ChIP-Chip, ChIP-Exo and Transposon Calling Cards (Supplementary File S4; see Section 2). We trained an XGBoost model to predict the probability of a potential edge being supported by evidence of direct binding. Since binding experiments are typically available for a limited number of TFs, we wanted to know if an XGBoost model trained on edges emanating from TFs that have binding data could predict probabilities of binding support for edges from TFs without binding data. Therefore, we trained and evaluated the model with 10-fold CV by TF; each hold-out fold consisted of edges from TFs that were not in the training fold. Throughout this article, we use 20 different random seeds for assigning TFs to CV folds and report the average performance. We trained and evaluated methods using two independent gene expression datasets for yeast that were generated by separate labs under different growth conditions. In each dataset, most TFs in the yeast genome were perturbed by either gene deletion (Kemmeren *et al.*, 2014) (TFKO data), or transient over-expression in response to a chemical inducer (Hackett *et al.*, 2020) (ZEV data).

We used independent, systematic, objective metrics for evaluating network maps, which we call *Binding*, *GO*, *GO-directness* and *PPI*:

- The binding metric is the fraction of highly scored edges that are supported by binding location experiments suggesting that the TF binds in the regulatory DNA of the target.
- The GO metric is based on over-representation analysis of the predicted targets of each TF. For each TF, the negative log *P*-value of the most significant GO biological process term for that TF’s targets is calculated. The median is reported, considering only TFs that have a significant GO term (adjusted *P* < 10^−4^). A higher score implies that the targets are more consistent in their biological functions.
- For each TF, the GO-directness metric considers only targets that are annotated with that TF’s most significant GO term. The metric is the fraction of those targets that are supported by binding data. We report the median of percent binding support across TFs. This metric might look like the binding metric, but it is distinct because it focuses exclusively on targets that contribute to identifying the TF’s main biological function.
- The PPI metric is the fraction of (TF, TF) pairs, with similar target sets, that are supported by evidence of physical interaction, and hence may function as a complex. Good performance on the PPI metric indicates that the network can predict experimentally verified biological facts, PPIs, which are independent of the data types used to construct the network. Specifically, we used a threshold on the scores of the TF network map to produce a discrete edge set and then calculated the Jaccard similarity between the predicted target sets of each pair of TFs (the size of the intersection divided by the size of the union). Next, we ranked all (TF, TF) pairs from highest Jaccard similarity index to lowest. The PPI metric is the fraction of (TF, TF) pairs with high Jaccard similarity for which there is strong evidence for a physical interaction in the STRING database (STRING score ≥ 0.7) (Szklarczyk *et al.*, 2021) (see Section 2 for details on each metric).

We expect the true yeast network to be sparse, with 10–50 targets per TF on average, so we focused our analysis on the top scoring edges. Before plotting, the *x*-axis was rescaled by the number of TFs scored, yielding the average number of targets per TF (see Supplementary Fig. S5).

First, we evaluated the NetProphet3 output score in comparison to its input scores from DE, LASSO, BART and PWM. Some of the input scores performed well on some metrics on one of the datasets, but overall, the NP3 synthesis was the clear winner (Supplementary Fig. S1A–H). Next, we compared NP3 to other prominent network mapping algorithms: NP2 (Kang *et al.*, 2017), Inferelator3 (Gibbs *et al.*, 2022; Jackson *et al.*, 2020), Genie3 (Huynh-Thu and Geurts, 2018) and a co-expression network based on the Spearman correlation between expression levels of the TFs and those of the genes. At each threshold, NP3 edges had a higher percentage of direct binding support than any of the others (Fig. 1B;Supplementary Fig. S2A and E show results broken out by TFKO and ZEV datasets; and Supplementary Fig. S7A and B show results using AUROC and AUPRC, respectively). On the GO metric, NP3 performance was about the same as Inferelator3 and Genie3 (though more consistent across thresholds), slightly better than NP2 (Fig. 1C and Supplementary Fig. S2B and F). Next, we calculated the GO-directness metric to check whether each algorithm’s performance on the GO metric was driven by direct edges, or came at the cost of including unwanted, indirect edges. NP3 targets annotated with the most significant GO term had higher binding support than those of the other algorithms (Fig. 1D and Supplementary Fig. S2C and G). By the PPI metric, NP3 performed better than any of the other expression-based methods, except that NP2 did slightly better at thresholds 60, 70 and 80 (Fig. 1E and Supplementary Fig. S2D and H). Considering all metrics, NP3 was the most accurate method tested.

### 3.2 The importance of TF-perturbation response data

**Fig. 2.**
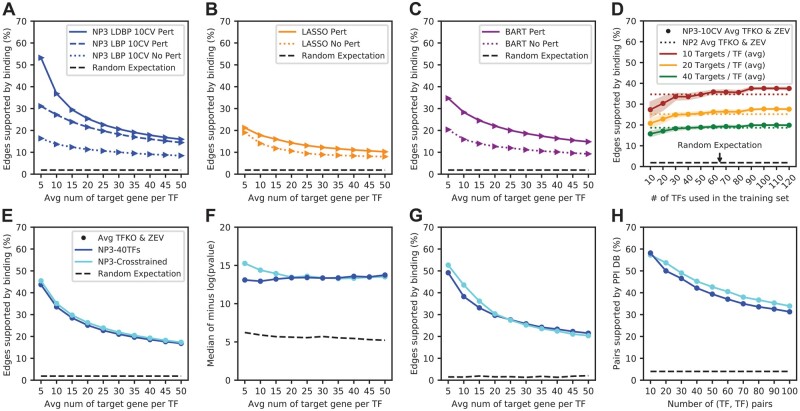
(**A**–**C**) Binding support for predicted edges without TF-perturbation profiles. (**A**) Solid line: NP3 run on TF deletion profiles from the TFKO dataset; dashed line: NP3 without the DE evidence score run on TF deletion profiles from the TFKO dataset; dotted line: NP3 without the DE evidence score run on an equal number of kinase deletion profiles from the TFKO dataset. (**B**) Evaluation of the raw LASSO evidence score on the TF deletion or kinase deletion expression profiles. (**C**) Evaluation of the raw BART evidence score on the TF deletion or kinase deletion expression profiles. (**D**–**H**) Performance of NP3 when little or no binding data are available (average of TFKO and ZEV datasets). (**D**) NP3 is trained with different numbers of TFs ranging from 10 to 120 and evaluated with the binding metric. Colors: three representative thresholds on the number of edges included in the network. Colored dotted lines: Accuracy of NP2, which does not require training, using the thresholds indicated. Shading: 1 SD of 20 networks trained on randomly selected subsets of TFs. Improvement is steepest up to 30 TFs of training data, matches NP2 at 40–50 TFs (depending on threshold), exceeds NP2 at 50–60 TFs, and plateaus at 90 or more TFs. Variability across randomly sampled training TFs also decreases as the sample size increases (shaded regions). (**E**–**H**) Binding, GO, GO-directness and PPI metrics, respectively. Comparison of training and testing on features derived from the same expression dataset (TFKO or ZEV) to training on one expression dataset and testing on another. In all cases, different TFs are used for training and testing. Dark blue: NP3 trained with 40 TFs and tested on different TFs using features derived from the same expression dataset. Cyan: NP3 trained by 10-CV with on features derived from one dataset and tested with features derived from another (‘cross-trained’). NP3 cross-trained on many TFs has a similar performance to NP3 on 40 TFs using the same expression dataset. Thus, using pre-trained parameters from a different dataset is better than retraining for a new dataset unless 40 TFs or more are available for retraining

NP3 can only use the DE score when TF perturbation data are available, but it can use the regression scores from any expression data. We wanted to understand how the DE component and the expression profiles of TF perturbations affected the performance of NP3. Using the TFKO dataset, we ran NP3 as usual and reran it without the DE score (Fig. 2A, solid and dashed lines, respectively). Excluding the DE score reduced the performance of NP3, suggesting that the DE score includes useful information that was not captured by LASSO or BART, even though LASSO and BART were run on the same TF perturbation profiles. Next, to estimate the performance of NP3 when TF perturbation profiles are not available, we reran it without the DE score, but we also replaced TF perturbation profiles with expression profiles for non-TF perturbations from the same dataset (Fig. 2A, dotted line). The performance of NP3 dropped further. LASSO and BART scores also became less useful (Fig. 2B and C). In addition to their value for generating a DE score, TF perturbation profiles also improve the performance of regression algorithms such as LASSO and BART. When these profiles were not available, the performance of LASSO and BART was comparable to that of NP3.

### 3.3 Using NetProphet3 when little to no binding data are available

We were able to train NP3 on many TFs that have binding data because *S.cerevisiae* is a well-studied organism. Less well-studied organisms have binding data on fewer TFs, so we investigated whether NP3 can be trained effectively on fewer TFs. We used TFKO and ZEV datasets and built separate networks for each. For each dataset, we trained NP3 on binding data from 10, 20, 30, …, up to 120 TFs. Each experiment was repeated 20 times with randomly selected TFs. Although we used different numbers of TFs for training, we predicted networks with the same number of edges by nesting random sub-sampling of TFs within the training folds of 10-fold CV. The performance of NP3 continued to improve as the number of training TFs increased, up to 90, after which it did not change (Fig. 2D). Training with fewer than 40 TFs yielded lower average performance than NP2, which has fixed parameters.

Next, we investigated whether a model trained on the ZEV dataset could make accurate predictions on the TFKO data, and vice versa (‘cross-trained’ in Fig. 2E–H; Supplementary Fig. S8A and B show results using the AUROC and AUPRC, respectively). By all metrics, the performance of NP3 cross-trained was at least as good as NP3 performance when trained on 40 TFs using features from the same expression dataset. Despite the differences between TFKO and ZEV datasets, which include growth format (batch versus continuous flow), nutrients and perturbation mechanism, parameters from one dataset transferred well to the other. Based on this transferability, we recommend using the model trained on yeast when applying NP3 to organisms that have binding data on < 40 TFs, to make predictions for the TFs that do not have binding data. Yeast models are provided as part of the NP3 distribution. Section 3.7 provides specific recommendations for mapping the targets of TFs that do have binding data.

### 3.4 Interpretable probability scores

**Fig. 3.**
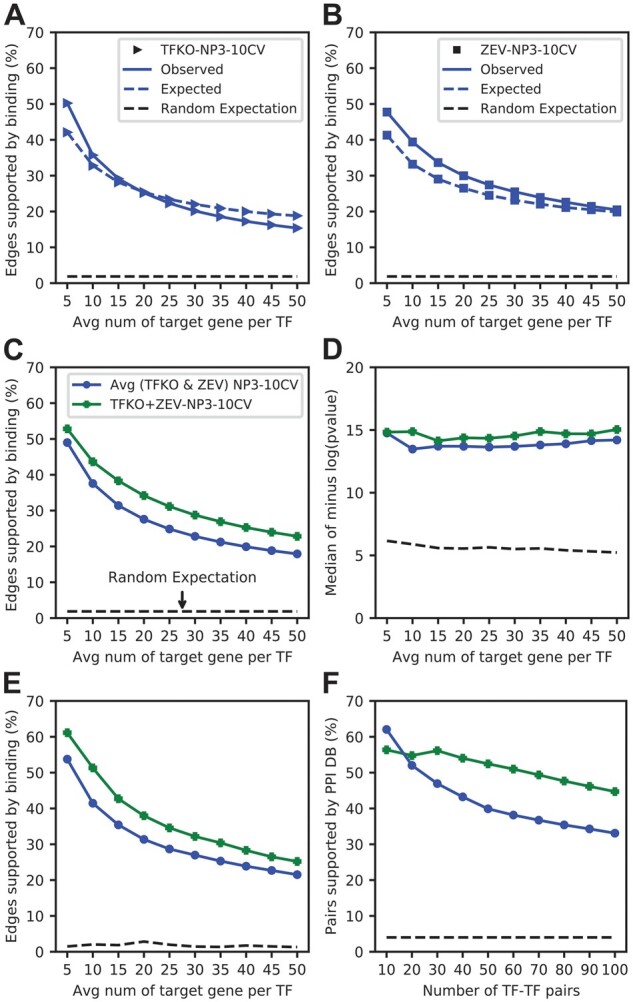
(**A** and **B**) NP3 edge predictions are interpretable as the probability of an edge being supported by binding data. Solid line: actual percent of predicted (TF, target) edges supported by binding data (observed); dashed line: average of predicted probabilities of (TF, target) edges multiplied by 100 (expected), as a function of the number of top-scoring edges in the network. (**A**) TFKO dataset. (**B**) ZEV dataset. The observed and expected lines are similar, but when they differ the observed fractions of binding support is usually higher than the expected. (**C**–**F**) Performance of NP3 framework when evidence scores from both TFKO and ZEV datasets are provided to XGBoost during training (eight features). Green line: NP3 trained with eight features (TFKO + ZEV). Blue line: Average performance of NP3 trained separately on four features from TFKO and four from ZEV. (**C**) Binding metric. (**D**) GO metric. (**E**) GO-directness metric. (**F**) PPI metric. By all four metrics, performance when training on features from both datasets exceeds that when each dataset is used separately

NP3 predicts the probability that a given edge will have a positive label, i.e. that it would be supported in a binding experiment. To explore whether these probability scores are accurate estimates, we ran NP3 and calculated the expected fraction of support by binding data (mean of predicted probabilities) for edges above various thresholds (Fig. 3A and B). The expected fractions were close to the actual fraction of edges supported by binding data; where they differed, the actual fraction usually exceeded the expected value. We can thus interpret NP3 output for an edge as the probability that the edge will be supported by evidence of direct binding. (The accuracy of the predicted probabilities when training on one dataset and testing on another is lower, Supplementary Fig. S3A and B.)

### 3.5 NetProphet3 can combine any number of evidence scores

One advantage of NP3 over NP2 is that it can use any number of evidence scores as features during XGBoost training. To test this out, we generated DE, LASSO, BART and PWM scores separately from the TFKO and ZEV datasets and provided all eight features to XGBoost during training and testing by 10-CV. By all metrics, the resulting network was better than using each dataset separately (Fig. 3C–F; Supplementary Fig. S9A and B show results using AUROC and AUPRC). The resulting network is provided as Supplementary File S8.

Next, we tried adding the scores output by another network mapping method, Genie3, as a fifth feature in separate analyses of the TFKO or ZEV datasets. This had essentially no effect on performance (Supplementary Fig. S4A–D), which shows that combining more network inference algorithms does not necessarily improve performance. To test how robust the framework is to low-quality features, we added scores from the Spearman correlation network, which is by far the worst of those we tested. Again, there was no effect on performance, showing that the framework is robust against low quality features (Supplementary Fig. S4E–H).

### 3.6 Discovery of potential new functions for TFs

So far, we have used the yeast data as a benchmark for developing and evaluating network mapping algorithms. Here, we shift from evaluation to exploration of the yeast network map. Since our purpose is to construct a highly accurate map, not to benchmark, we trained NP3 on all the binding data rather than using CV. We trained NP3 using the eight features from TFKO and ZEV datasets together and thresholded the network at an average of 25 targets per TF (Supplementary File S9). We then investigated two aspects of the resulting network. First, we considered pairs of TFs with highly overlapping target sets as predicting PPIs between the TFs. Second, we considered the GO biological process term that was most enriched among the targets of a TF as predicting the function of that TF.

#### 3.6.1 TFs with the most similar NP3-predicted target sets form well-studied complexes that are known to regulate the TFs’ shared targets

**Fig. 4.**
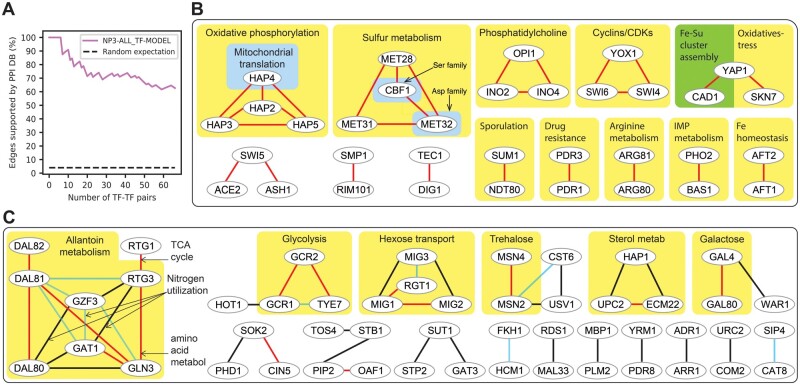
Exploration of the NP3 network trained using the eight features from TFKO and ZEV datasets together and all binding data (without CV), thresholded at an average of 25 targets per TF. (**A**) The PPI metric at the resolution of one (TF, TF) pair, for pairs with Jaccard similarity >0.1. (**B**) Connected components of the (TF, TF) network containing edges that connect TFs with Jaccard similarity >0.1 and more than one target in common. Examples of common functions of connected TFs are indicated. Connected components whose edges are all strongly supported (STRING score ≥ 0.7). (**C**) Same as (B) showing all connected components with at least one edge that is not supported by strong PPI evidence. Red: Strong PPI support. Blue: Moderate PPI support (0.4 ≤ STRING score < 0.7). Black: Weak or no PPI support (STRING score < 0.4)

Figure 4A shows the PPI evaluation metric for this network at a resolution of one TF pair, for all 67 (TF, TF) pairs with Jaccard similarity above 0.1. The nine (TF, TF) pairs with the highest Jaccard similarity all interact physically and 62% of pairs with similarity above 0.1 do. To investigate further, we formed an undirected network in which TFs are vertices and two TFs are connected if they share more than one target in common and the Jaccard similarity of their target sets is >0.1. Figure 4B and C show the connected components of this graph. Most edges are supported by strong evidence for physical interaction according to the STRING database (0.7 ≤ score, red) and most of the rest are supported by moderate evidence (0.4 ≤ score < 0.7, cyan). Only 23 edges had STRING score <0.4, indicating little or no evidence of physical interaction in STRING. Thirteen out of 29 connected components have all edges strongly supported (Fig. 4B and C). This includes two four-member components, both of which correspond to well-documented complexes including Hap2/3/4/5 and Met28/Met31/Met32/Cbf1. Edges connecting Met28 and Met32 to Met4, another known interactor, scored just below the 0.1 threshold for inclusion. GO over-representation analysis on the shared, predicted targets of connected TFs revealed shared functions corresponding to the known biological roles of the complexes. For example, all edges in the well-documented Hap2/3/4/5 complex showed enrichment for shared targets involved in oxidative phosphorylation, the known function of the complex (Boos *et al.*, 2019) (Hap1 is not functional in *S.cerevisiae* S288C (Gaisne *et al.*, 1999)). Similarly, all edges in the Met28/Met31/Met32/Cbf1 component showed enrichment for common targets involved in sulfur and methionine metabolism, the known function of these complexes (Petti *et al.*, 2012).

The largest connected component consists of eight TFs (Fig. 4C) that are known for their roles in nitrogen utilization and amino acid metabolism (Ljungdahl and Daignan-Fornier, 2012). Six edges have strong evidence of physical interaction from STRING and five have moderate evidence. The high Jaccard similarity of these five edges supports this moderate evidence and makes it likely that these pairs indeed interact physically. Four edges are not currently supported by STRING, but one of them, Gzf3-Dal80, is supported in the literature (Svetlov and Cooper, 1998). The high Jaccard of the remaining three suggests that they may interact physically. Most of the TF pairs share targets that are enriched for metabolism of allantoin, a nitrogen source, or the more general processes of nitrogen utilization or amino acid metabolism, consistent with the known functions of these TFs. The common predicted targets of Rtg1 and Rtg3, a well-documented complex, are involved in the TCA cycle, which is the starting point for many amino acid metabolism pathways (Ljungdahl and Daignan-Fornier, 2012).

#### 3.6.2 NetProphet3 highlights potential novel functions of TFs

Next, we carried out over-representation analysis on the predicted targets of each TF in the NP3 network map. For most TFs, the most significantly enriched biological process term and the target genes annotated with it corresponded to a well-documented function of that TF (not shown). However, there were several cases where it represented a potential novel function. For example, Nrg1, known primarily for its roles in glucose repression (Park *et al.*, 1999) and repression of the alkaline pH response (Lamb and Mitchell, 2003), was predicted to regulate targets that are heavily enriched for genes involved in iron ion transport. A connection between Nrg1 and iron homeostasis had been reported in *Candida albicans* (Moran, 2012; Murad *et al.*, 2001) but not, to our knowledge, in *S.cerevisiae*. Although these genes were not bound by Nrg1 according to the curated binding network we used for training NP3, unpublished experiments using the Calling Cards method show that Nrg1 does bind to four of these: *FIT2*, *FIT3*, *FTR1*, *SMF1* (Rob Mitra, personal communication). Thus, Nrg1 directly regulates iron transport in *S.cerevisiae*. Similarly, the predicted targets of Rtg3, which is known for its role in regulating the TCA and glyoxylate cycles in response to mitochondrial dysfunction, were highly enriched for genes involved in synthesis of glutamine family amino acids. Some of these genes are previously reported functional targets (*IDH1*, *IDH2*) (Liu and Butow, 1999) and three were found to be bound by Rtg3 in a ChIP experiment (*ARG3*, *CPA2*, *IDH2*, *IDP1*) (MacIsaac *et al.*, 2006), but none of them were present in the binding network we used for training. Thus, Rtg3 directly regulates genes involved in biosynthesis of arginine, which feeds into synthesis of other glutamine family amino acids.

### 3.7 Integration mode: using NetProphet3 to forge consensus networks from gene expression and binding data

**Fig. 5.**
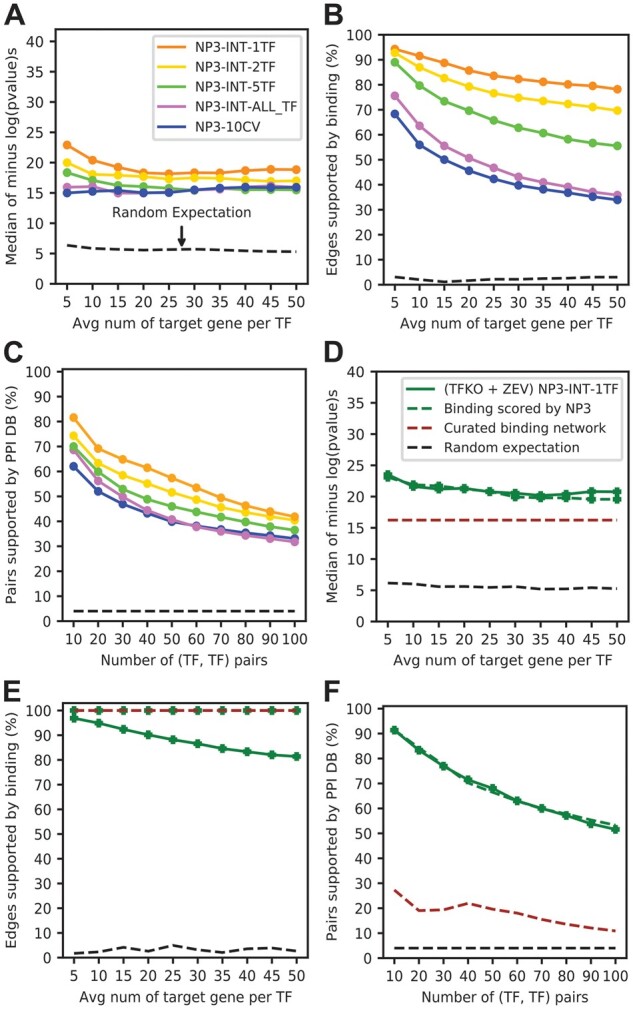
(**A**–**C**) Performance of NP3 in integration mode for TFs that have binding data. Gene expression and binding data are integrated by using NP3 predictions on the same edges it was trained on (average of the TFKO and ZEV datasets). (A–C) Blue: NP3 10-CV by TF. Purple, lime, gold and orange: models trained on all, 5, 2 or 1 TF, respectively. (A) GO metric. (B) GO-directness metric. (C) PPI metric. By all metrics, NP3 in integration mode (NP-INT) outperforms NP3 in generalization mode (NP3-10CV). In integration mode, performance improves as fewer TFs are used in the training. (**D**–**F**) Performance of NP3 in integration mode compared to the curated, binary binding network used in training (TFKO and ZEV are combined into eight features). Green solid: NP3 in integration mode; brown dashed: positively labeled edges from the training data; green dashed: positively labeled edges from the training data, ranked by NP3 scores (Method 2 in text). (D) Evaluation with the GO metric. (E) GO-directness. (F) PPI metric. NP3 in integration mode outperforms the curated binary binding network that was used to train it, by the GO and PPI metrics, but the binding network has, by construction, 100% of edges supported by binding data. When the binary binding network is scored by NP3 predictions, binding support remains 100% but the performance with GO and PPI is greatly improved

So far, we have shown that NP3 can be trained on TFs that have binding data to predict target probabilities for TFs without binding data. In this section, we show that NP3 can forge an even better network for TFs that have binding data by integrating the gene expression and binding data. For the previous section, we predicted edges using eight features (from TFKO and ZEV) by training one model with all available TFs that have binding data, rather than by CV. We wondered whether performance would degrade when a limited number of TFs is available for training, so we trained with fewer TFs. We did not evaluate these networks with the binding metric because predictions were not blind to binding data. Unexpectedly, all other metrics showed that training on fewer TFs improved performance (Fig. 5A–C). The best performance was achieved by training a separate model for each TF and making predictions on the same data used in training. Predicted edge probabilities from these TF-specific models were combined to form a single weighted network. Substantial improvements were achieved by overfitting models to binding data for a specific TF. To check whether these improvements are due to integrating the expression data with binding data, rather than simply memorizing the binding data through overfit, we randomized the expression features and then repeated the single-TF training and evaluation. Randomization of expression features greatly reduced performance, showing that good performance does require integration of expression and binding data (Supplementary Fig. S11 and Supplementary Section S7). Traditionally overfitting is avoided, so trained models can generalize on unseen data. Here, the goal of the model was not to make predictions on unseen data but rather to integrate the expression data (features) with the binding data (labels) to produce a consensus network. Overfitting to a single TF’s data increased the influence of binding data on the predictions, resulting in a more effective integration of gene expression and binding data, and hence a more accurate TF network map according to evaluation metrics that are independent of the training data. We refer to this application of NP3 as integration mode and the original application as generalization mode.

For integration mode to be useful, the NP3 predictions must be better than the labels used in training—otherwise, one would simply use the labels directly. The binary training labels were derived by curation from a variety of datasets generated with different technologies, so they did not have any associated confidence scores. For a binary network, the only threshold that can be applied includes all edges with score 1, so we evaluated the positively labeled edges. NP3 scores were much better than the binding network by the GO and PPI metrics (Fig. 5D–F, solid green versus dashed brown). Among NP3-predicted targets with the most significant GO term for each TF, most were supported by binding data. But for the binding label network, by definition, all targets are presumed direct. This suggested another way to use NP3 in integration mode: use NP3 to rank the edges labeled 1, so that multiple thresholds can be applied to them, while leaving the edges labeled 0 with score 0. This can be viewed as constraining the NP3 scores for 0-edges, but not for 1-edges. Ranking the binary network in this way improved it greatly, according to both the GO and PPI metrics (Fig. 5D–F, dashed green vs. dashed brown). In fact, the performance of the constrained NP3 scores was comparable to that of the original NP3 scores in integration mode at every threshold. However, all edges with non-zero score are now supported by binding data.

Throughout this article, the binding labels we used were binary—since they were derived from multiple datasets and experimental methods, they could not be directly compared. However, individual binding datasets often come with *P*-values or other confidence scores. The largest such dataset for yeast is the comprehensive ChIP-Chip data (Harbison *et al.*, 2004). We considered two ways of using this binding dataset to rank network edges. Method 1 is to simply rank the edges from most significant *P*-value to least significant. Method 2 is to choose a threshold on *P*-values, assign all edges below the threshold a score of 0 (bottom of ranking), and rank the edges above threshold by their NP3 scores. NP3 was used in integration mode with the 3% of most significant ChIP edges labeled positive and the remainder labeled negative. Both Method 1 and Method 2 guarantee that all the top scoring edges are supported by ChIP data. To compare them, we used the GO metric and the PPI metric, which are independent of binding data. The overall performance was lower than when training on the curated network, likely due to lower-quality binding data. However, thresholding the ChIP-Chip *P*-values and rescoring the edges above threshold with NP3 greatly improved the results, compared to ranking the positive edges using their original *P*-values from ChIP-Chip (Supplementary Fig. S6). Again, all edges in both networks are presumed direct based on the ChIP-Chip data. Thus, re-ranking edges of a binding dataset with NP3, run on a TF perturbation-response expression dataset, can improve the network without adding indirect edge.

### 3.8 Availability and implementation

NetProphet3 is also available on GitHub: https://github.com/BrentLab/NetProphet_3.0. The version that we used in this article is 3.0. The instructions on how to use the package are in the README file.

## 4 Discussion

NP3 exploits a supervised machine learning framework in order to predict the direct, functional targets of a TF from gene expression data. The features are built from gene expression datasets by a combination of linear- and tree-based regression algorithms and DE analysis (Fig. 1A). However, the framework is general enough to incorporate any number of features, such as edge scores from other network mapping algorithms or from scanning TF binding-specificity models across each gene’s regulatory DNA. The binary training labels are constructed from TF-binding location data, with 1 indicating that the available data suggests the TF binds in the cis-regulatory DNA of the target. This approach reflects our goal, which is to find direct, functional targets of each TF. The system learns to recognize the patterns in the gene expression data that are characteristic of directly bound targets. Binding datasets are far from perfect and, even when a TF does bind a gene’s regulatory DNA, it may not regulate that gene. However, it is unlikely that NP3 would predict targets that are bound but not functional, since its predictions are based on gene expression features that reflect functional regulation.

NP3 can be used to predict direct, functional targets of TFs for which gene expression data, but not binding data, are available. When working with an organism for which at least 40 TFs have binding data, NP3 can be trained specifically for the gene expression and binding datasets from that organism. When working with an organism for which few TFs have binding data, NP3 models trained on yeast data can be used. In this application, the XGBoost machine learning framework is being used in the typical way, to generalize from training data (TFs with binding information) to previously unseen test data (TFs without binding information). To test NP3’s accuracy in this application, we developed three systematic, large-scale evaluation metrics: one based on binding data (a direct test of generalization), one based on GO biological process terms and one based on the PPI network. The GO metric is an indirect test of functional regulation, since there is no reason to expect non-functional binding sites to be biased toward genes involved in the same biological process as functional binding sites. NP3 was clearly more accurate than the other leading algorithms we tested on the binding- and PPI-based metrics. NP3, Inferelator3 and Genie3 had similar performance with the GO metric (Fig. 1C). However, even among the target genes annotated with the most enriched GO term, Inferelator3 and Genie3 predicted targets were much less likely to be direct than NP3-predicted targets.

A limitation of NP3 is that its good performance depends on the availability of gene expression data after direct TF perturbations. Unlike purely regression-based algorithms, NP3 benefits greatly from DE analysis (Fig. 2A–C). In addition, the regression methods that NP3 uses, LASSO and BART, also receive a boost in accuracy from TF perturbation data, even though they do not use any information about which TF was perturbed in each sample. That is likely because direct TF perturbations increase the variability of the TF expression levels in the dataset, making it easier to detect correlations between the expression levels of TFs and their targets. This suggests that other regression-based algorithms will also perform better with TF perturbation data than without.

For TFs that have both perturbation-response data and binding location data, NP3 can be used to forge a consensus that integrates these two data types. In this application, a separate XGBoost model is trained for each TF. The predictions of this model are then used to score the same (TF, target) edges that were used in training. Here, XGBoost is not being used for generalization to data not seen during training, but rather to integrate the expression datasets, from which the features are derived, with the binding datasets, from which the labels are derived. The binding-based labels are used only during training, but because the model is intentionally overfit to them, their influence is incorporated into the model and hence the predictions. In this way, NP3 becomes a tool for multi-omics integration. In integration mode, NP3 trains on all the binding data, rather than using CV to test generalization to unseen data, so binding data cannot be used to evaluate it. However, the GO and PPI metrics can still be used. These metrics show that the multi-omics integration mode outperforms predictions based only on gene expression data (Fig. 5A–C). Furthermore, NP3 scores outperform the network defined by the binary training labels or the one defined by the minus Log_10_*P*-value of ChIP-Chip experiments. Integration mode can also be used to produce a network map whose edges are all supported by binding data. Here, the NP3 scores are used only to re-rank edges with binding support, setting the scores of all other edges to zero. Constraining NP3 in this way produces a network in which all edges are direct without degrading performance.

This novel application of machine learning can be generalized to other tasks in multi-omics integration. The fundamental idea is to derive features from some of the omics modalities and labels from a different modality. Each modality typically contains considerable technical variability (‘noise’). In the first phase, a machine learning model is fit (potentially overfit) to the training data. In the second phase, the model is used to make predictions on the same instances used in training. The labels are used only in the first phase, but they exert an influence on the second-phase predictions through the learned model. These second-phase predictions consist of a single number for each instance that integrates the omics modalities used to construct the features and the labels for that instance. These numbers can be thought of as a consensus of the noisy labels and noisy features.

## Supplementary Material

btad038_Supplementary_Data
