# Supplementary material for: NetProphet 3: a machine learning framework for transcription factor network mapping and multi-omics integration
Source: Bioinformatics. 2023 Jan 24;39(2):btad038. doi: 10.1093/bioinformatics/btad038 (PMC9912366; doi:10.1093/bioinformatics/btad038)
Supplement: btad038_Supplementary_Data [file btad038_supplementary_data.pdf]

Supplementary files

Files are available at: https://zenodo.org/record/7504131#.Y7Wu3i-B2x8

File. S1. (A-B) tfko_expr_6112_1485_indexed.tsv, zev_expr_6175_591_indexed.tsv

Gene-expression ($\log_2$ fold-change) for all genes for the TFKO and ZEV datasets. Rows and columns are indexed by the list of target genes and sample ids, respectively. Sample id is the systematic name of the perturbed TF, if the sample is a TF perturbation profile.

File. S2. (A-B) tfko_expr_reg_313_1485_indexed, zev_expr_reg_320_591_indexed

Gene-expression ($\log_2$ fold-change) for TFs for the TFKO and ZEV datasets. Rows and columns are indexed by the list of TFs and sample ids, respectively. Each File is a subset of the corresponding gene-expression file of the target genes.

File. S3. (A-B). tfko_de_logfc_313_6112_indexed, zev_de_shrunken_320_6175_indexed

DE weighted networks for the TFKO and ZEV datasets. Row and columns are indexed by the list of TFs and target genes. Each score is the log2 fold-change of the target gene after the perturbation of that TF. Scores between 1.5 and -1.5 are replaced by zero.

File. S4. eval_reg_target_cc_exo_chip_exclusive.tsv

Curated list of edges that have evidence of direct binding and used in training NP3 and binding evaluation metric. The first column is for regulators and the second column is for targets.

File. S5. eval_ppi_STRING.txt

Protein-protein interaction collected from the STRING database v.11.5 used in the PPI evaluation metric. The first two columns are the names of the interacting proteins, and the third column is the confidence score (a probability score) multiplied by 100.

File. S6. eval_gene_association.sgd

This file is used by the GO-Term-Finder v.0.86 package for GO enrichment evaluation. It encodes the association between GO terms and genes, and it is downloaded from http://geneontology.org/.

File. S7. eval_gene_ontology_edit.obo

This file is also used by the GO-Term-Finder v.0.86 package for GO enrichment evaluation. It includes a list of GO terms for Saccharomyces cerevisiae and it is downloaded from http://geneontology.org/

File. S8. output_np3_network_tfko_zev_by_10cv.tsv

A NP3 network generated by 10-CV and by combining the TFKO and ZEV datasets. LASSO, DE, BART, and PWM were generated for each dataset making a set of eight features. There are three columns for regulators, targets and NP3 probabilities, respectively.

File. S9. output_np3_network_tfko_zev_by_integration_all.tsv

A NP3 network generated by integrating gene-expression and binding data and combine the TFKO and ZEV datasets. Integration is done by training one single model using all TFs. There are three columns for regulators, targets and NP3 probability scores, respectively.

File. S10. output_np3_network_tfko_zev_by_integration_tf1.tsv

A NP3 network generated by integrating gene-expression and binding data and combining the TFKO and ZEV datasets. Integration is done by combining predictions from models, each trained by edges of one single TF. There are three columns for regulators, targets and NP3 probabilities, respectively.

File. S11. output_curated_binding_network_scored_np3_integration_tf1.tsv

A TF network map generated by ranking the positive edges from the curated binding network (in file eval_reg_target_cc_exo_chip_exclusive.tsv) by NP3 probability scores (in file output_np3_network_by_integration_tf1.tsv). It is considered the best network possible with the current available datasets.

File. S12. output_most_significant_go_term_for_curated_binding_scored_by_np3_integration_tf1.tsv

Each TF in the network (in the file output_curated_binding_network_scored_np3_integration.tsv) is assigned the GO term with the highest minus Log10 P-value across thresholds from 5 to 50 targets per TF, on average. Columns are as follows: TF, GO id, short description of the GO term, P-value, fold enrichment, list of target genes in the network that matched that GO term, threshold number.

NetProphet_3.0.zip

NetProphet 3.0 package, which includes scripts, data files for TFKO and ZEV models.

Supplementary figures

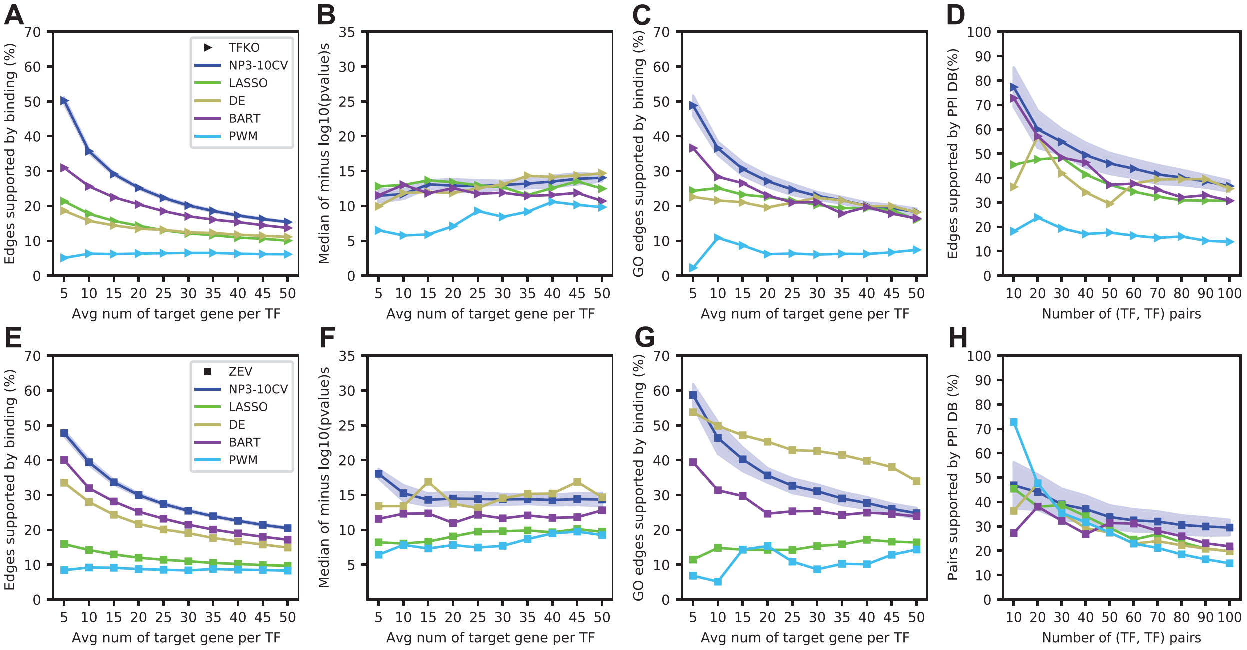

Fig. S1. (A-H) Performance of NP3 compared to its features LASSO, DE, BART, and PWM. Blue: NP3 using 10-CV by TF; Blue shaded area: one standard deviation of 20 networks from different 10-CV seeds; lime: LASSO; olive: DE; purple: BART; turquoise: PWM. (A-D) and (E-H) networks predicted for TFKO and ZEV dataset. (A, E) Binding metric. NP3 outperforms substantially and consistently its combined evidence scores with both datasets. (B, F) edges are evaluated with the GO metric. NP3 is comparable to its features with TFKO dataset (B), but more consistent. NP3 outperforms substantially all intermediate evidence scores, except DE in thresholds 15, 35, 40, 45 with ZEV dataset. (C, G) GO-directness metric. NP3 outperforms all evidence scores except in later thresholds, they have similar performance. With the TFKO dataset (C). NP3 outperforms all evidence scores except DE in thresholds 15, 35-50 with the ZEV dataset. (D, H) PPI metric. NP3 outperforms all evidence scores with the TFKO dataset (D). NP3 outperforms all evidence scores except PWM in thresholds 5 and 10 with the ZEV dataset (H).

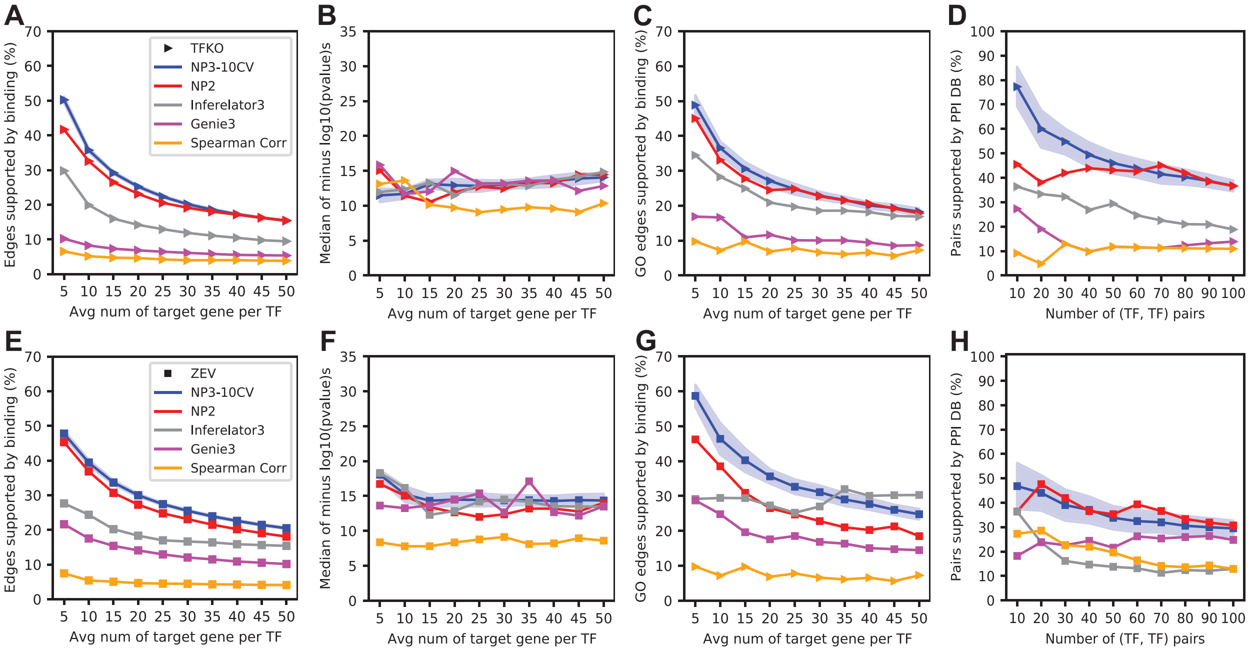

Fig. S2. (A-H) Performance of NP3 and other network inference algorithms using the binding evaluation, GO, and PPI metrics. Blue: NP3 using 10-CV by TF; Blue shaded area: one standard deviation of 20 networks from different 10-CV seeds; red: NP2 fixed parameters; gray: Inferelator3; magenta: Genie3; orange: Spearman correlation. (A-D) and (E-H) networks predicted by TFKO and ZEV dataset, respectively. (A, E) edges are evaluated with the binding metric. NP3 consistently outperforms its predecessor NP2, Inferelator3, Genie3, and Spearman correlation. (B, F) edges are evaluated with the GO evaluation metric. (B) with the TFKO dataset, NP3 and other network inference algorithms have comparable performance, though NP3 is more consistent. (F) with the ZEV dataset, NP3 outperforms other network inference algorithms, except for thresholds 10, 25 and 35. (C, G) edges matching the most significant GO term are evaluated with binding support. For each set of TF edges, binding support is calculated and average across TFs is reported. NP3 TF edges matching the most significant GO term have higher binding support than that of NP2, Inferelator3, Genie3, and Spearman correlation. (D, H) edges are evaluated using the PPI/Jaccard metric. (D) with the TFKO dataset, NP3 outperforms all other network inference algorithms except for thresholds 70 and 80. (H) with the ZEV dataset, a better performance than that of Genie3 and Spearman correlation, but not better than that of Inferelator3.

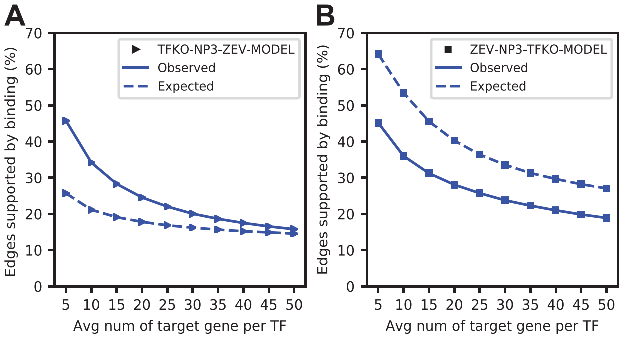

Fig. S3. (A-B) NP3 edge predictions are estimated probabilities of edges being supported by binding data. Solid line: actual percent of (TF, target) edges supported by binding data (observed); dashed line: average predicted probabilities of (TF, target) edges multiplied by 100 (expected), both as a function of the number of top-scoring edges in the network. (A) NP3 trained with the ZEV dataset to predict the TFKO dataset; the observed and expected binding support have similar trends, but the observed is much higher than the expected. (B) NP3 trained with the TFKO dataset to predict the ZEV dataset; the observed and expected binding support have the same trend, but the observed is much lower than the expected.

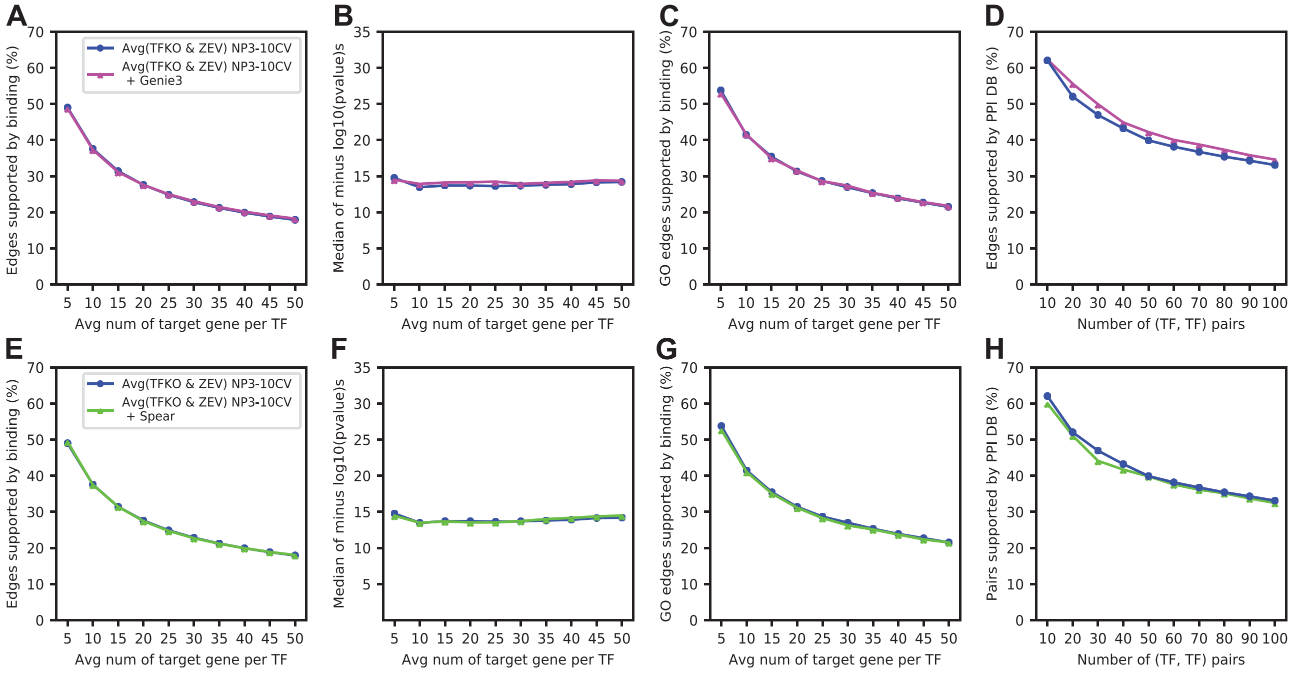

Fig. S4. (A-H) Performance of the NP3 when adding output of Genie3 or Spearman Correlation as a 5th feature, compared to the original NP3 using only DE, LASSO, BART, and PWM. (A-D) Pink: NP3 framework using Genie3 output; Blue: original NP3. (A) Performance on the binding metric. (B) Performance on the GO metric. (C) Performance on the GO-directness metric. (D) Performance on the PPI metric. The performance of the original NP3 and that with Genie3 is consistently similar across all independent evaluation metrics. (F-I) Same as A-D, respectively, except that the green line shows NP3 framework using Spearman correlation between the expression of TFs and targets as the 5th feature. Adding the low-quality Spearman correlation score does not degrade accuracy.

Threshold	(TF, gene)	NP3 scores	Binding support	% binding support
	(TF1, Gene02)	0.86	1	
	(TF1, Gene20)	0.85	0	
	(TF2, Gene01)	0.84	0	
	(TF1, Gene01)	0.80	1	
	(TF1, Gene04)	0.80	1	
	(TF1, Gene22)	0.80	0	
	(TF2, Gene03)	0.79	1	
	(TF2, Gene21)	0.79	0	
	(TF1, Gene32)	0.76	0	
	(TF1, Gene10)	0.75	1	
(5)				(50%)
	(TF2, Gene19)	0.70	0	
	(TF2, Gene09)	0.69	1	
	(TF2, Gene56)	0.69	0	
	(TF1, Gene89)	0.65	0	
	(TF2, Gene08)	0.64	0	
	(TF1, Gene11)	0.64	1	
	(TF1, Gene03)	0.60	0	
	(TF2, Gene22)	0.58	0	
	(TF2, Gene32)	0.58	1	
	(TF2, Gene10)	0.57	0	
(10)				(40%)

Fig. S5. Explanation of how thresholds are constructed for evaluation with the binding, GO, and GO-directness metrics. Scored edges are sorted from highest to lowest (column 3). In this example the network has only two TFs, so the first threshold, which is five targets on average, has the top 10 scoring edges (2x5=10). Notice that in this threshold, TF1 has greater number of targets compared to TF2 (seven targets compared to two targets). Next, the second threshold, ten targets on average, has the top 20 scoring edges (2x1=20). As mentioned in the main text, this method enables us to compare the performance of networks with different number of TFs, the number of targets are scaled the number of TF in each threshold. If a network has 100 TFs, the first and second thresholds will have 500 and 1000 edges respectively. Once thresholds are set, evaluation metrics can be calculated, here with the binding metrics the number of edges supported by evidence of direct binding are calculated as presented in the last two columns.

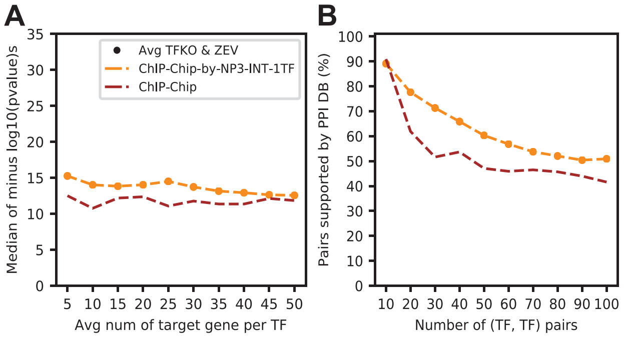

Fig. S6. (A-B) Performance of ChIP-Chip network scored by minus Log_{10} P-values from ChIP-Chip experiments vs. top 3% of ChIP-Chip edges scored by NP3 integration method and trained using 1 TF at a time. (A) GO metric and (B) PPI metric. Performance of ChIP-Chip edges ranked using NP3 integration method is better than that ranked using minus Log_{10} P-value from ChIP-Chip experiments.

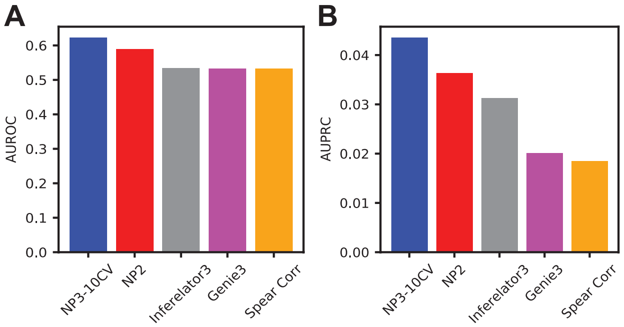

Fig. S7 (A-B) Performance of NP3, Inferelator3, Genie3, and Spearman Correlation (Spear Corr) using: (A) Area Under the ROC Curve (AUROC) and (B) Area Under of Precision Recall Curve (AUPRC). The AUROC and AUPRC are calculated using binding data. The Performance of NP3 is better than NP2, than Inferelator3, than Genie3, and then Spearman Correlation.

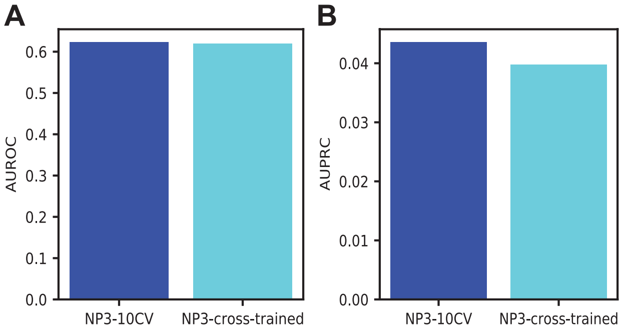

Fig. S8 (A-B) Performance of NP3-10CV and NP3-cross-trained using: (A) Area Under the Curve (AUROC) and (B) Area Under of Precision Recall (AUPRC). The AUROC and AUPRC are calculated using binding data. The performance of NP3-10CV and NP3-cross-trained are about equal.

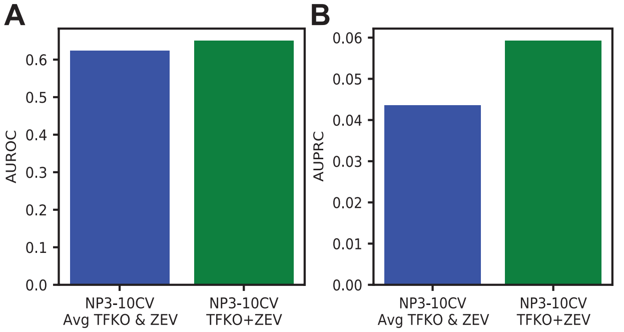

Fig. S9 (A-B) Performance of NP3-10CV using either TFKO or ZEV dataset. (A) Area Under the Curve (AUROC) and (B) Area Under of Precision Recall (AUPRC). The AUROC and AUPRC are calculated using binding data. The performance of NP3-10CV using combined TFKO and ZEV datasets is better than using either of TFKO and ZEV dataset.

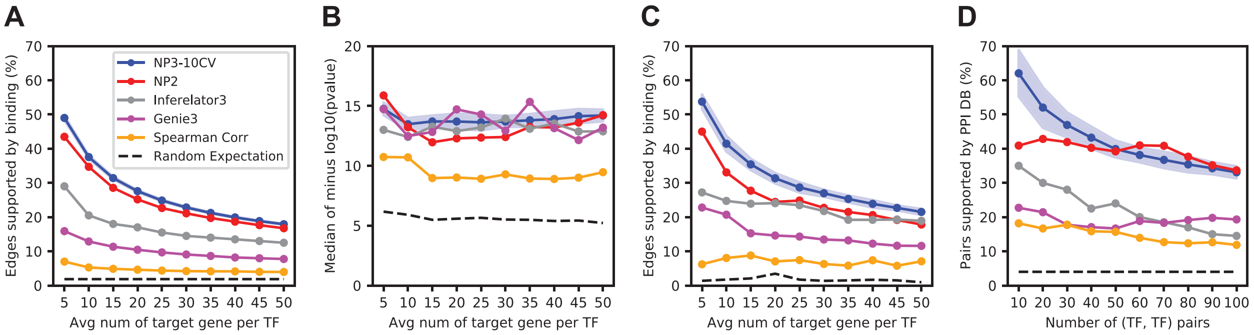

Fig. S10. (A-D) Performance of NP3, NP2, Inferelator3, Genie3 and Spearman Correlation using the metrics of binding (A), GO (B), GO-directness (C) and PPI (D). These figures are slightly different from Figure 1 in the main manuscript. The performance of NP3, NP2, Genie3 and Spearman Correlation is the same as Figure 1 in the main manuscript, but Inferelator3 is different. Here Inferelator3 was run with all TFs that have a DE prior and that do not have a DE prior. TFs that do not have a DE prior, a prior of zeros was used. Here, the performance of Inferelator3 is slightly lower than that of Figure1, which includes only TFs that have prior.

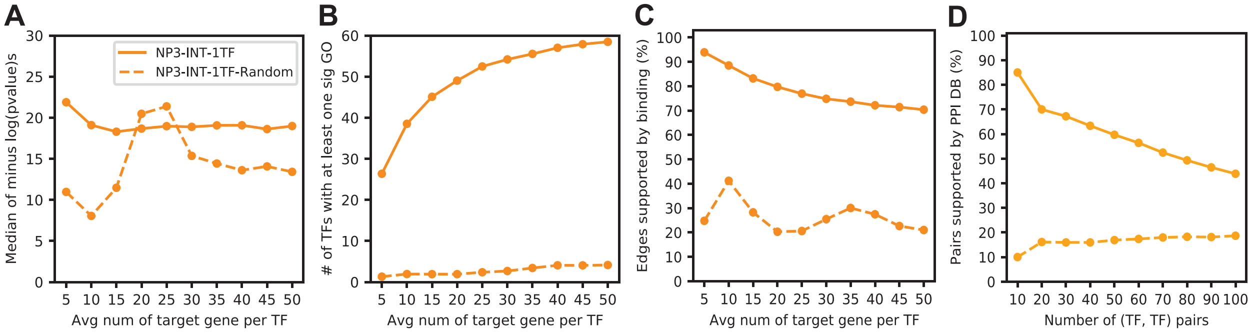

Fig. S11. (A-D) Performance of NP3 using the integration method by building a TF-specific model: original run (orange solid line) and randomized run (orange dashed line). The randomized run is done by permuting (TF, gene) edge scores of each of the feature LASSO, DE, BART, PWM. (A) Performance using the GO metric. The performance of the randomized run is much lower than that of the original run, except for thresholds 20, 25. (B) The number of TFs that have at least one significant GO term. The randomized run has less than 5 TFs compared to an increasing number of TFs that reaches 60 TFs by threshold 50 for the original NP3 run. (C) Performance using the GO-directness metric. (D) Performance using the PPI metric. Again, the randomized run has a much lower performance than the original NP3 run.

Supplemental methods

1. NetProphet3

In the experiments reported here, we used NP3 v1.0. The latest version of NetProphet3 can always be found on GitHub https://github.com/BrentLab/NetProphet_3.0. The training labels and input data are provided as supplemental files S1-4. NP3 combines four weighted networks LASSO, DE, BART, and PWM. Each one of them is inferred by different intermediate software. For LASSO, we used 10-fold CV to select lambda with the option of one lambda used for all targets. We used the R packages: Lars v1.2 for LASSO, BayesTrees v0.3.1.4 for BART and FIRE v1.1 for PWM as described in the NetProphet2.0 paper (Kang, et al., 2017). We used the weighted network output by BART as the input to FIRE. Learned PWMs were scanned over promoters using FIMO from the MEME suite. NP3 used packages XGboost v1.3.2.1 (Chen and Guestrin, 2016) and mlr v2.19.0 (Bischl, et al., 2016) for training and tuning hyperparameters.

2. Genie3

We used Genie3 available in this GitHub page https://github.com/vahuynh/GENIE3, we ran the R implementation with default parameters.

3. Inferelator3

We used Inferelator3 in this GitHub page https://github.com/flatironinstitute/inferelator and ran it with parameters regression="bbsr", workflow="tfa".

4. Spearman co-expression network

We calculated the spearman correlation between the expression profiles of each target gene - the first vector - and each regulator (TF) - the second vector. Each of the spearman correlation values is the score of the edge of that target gene and that TF.

5. Gene-expression profiles

We used two gene-expression datasets for Saccharomyces cerevisiae in which each TF was perturbed either by deletion (TFKO) (Kemmeren, et al., 2014) or overexpression (ZEV) (Hackett, et al., 2020). The TFKO dataset that we downloaded from http://deleteome.holstegelab.nl/data/downloads/deleteome_all_mutants_controls.txt and includes 1485 gene expression profiles of strains in which a single gene was deleted from the genome. In 281 of these strains a TF was deleted. We used the TF perturbation profiles to construct the DE features by replacing fold changes between -1.3 and 1.3 by 0. Values from the original dataset were negated so that positive values correspond to an activating edge and negative values correspond to a repressive edge. We used all 1485 gene expression profiles, unmodified, as input to LASSO and BART. We used the column labeled log2_cleaned_ratio of the ZEV downloaded file https://storage.googleapis.com/calico-website-pin-public-bucket/datasets/pin_tall_expression_data.zip. This dataset contains gene expression profiles at various time points after transient induction of 167 TFs using estradiol. Gene expression was measured at various time points after induction. To construct DE features, we used shrunken $\log_2$ fold-change data from 15-minutes after induction and replaced fold changes between -1.3 and 1.3 by 0. As input to LASSO, DE, and other regression algorithms, we used 591 gene-expression profiles time points 15, 45 or 90 minutes after induction. All inputs are provided as supplemental files S1-3.

6. Evaluation metrics

Code for evaluation metrics: binding, GO, GO-directness and PPI is available in one package and can be found on GitHub https://github.com/BrentLab/NET-evaluation. We note that the GO evaluation metric is used as follow. For each TF's targets, we did GO enrichment analysis using GO-Term-Finder v0.86 (Boyle et al. 2004). We downloaded GO biological process terms from the gene ontology website http://geneontology.org/. We used the downloaded files to replace the annotations that came with the GO-Term-Finder v0.86. We provided these files supplemental files S6-7.

7. Overfitting a TF-specific model with a randomized feature set

We randomly permuted (TF, gene) edge scores for each of the feature LASSO, DE, BART, and PWM. Then, we ran NP3 by overfitting a TF-specific model (integration mode). Of the top 5000 edges, 4996 were from one TF, STE12, in the TFKO data, and 4993 were from STE12 in the ZEV data. Since STE12 was such an outlier, we removed it reran the experiment. Next, we evaluated the network with the GO, GO-directness and PPI metrics. Results show that the network predicted from randomized features had much lower performance than NP3 without randomization (Supplementary Fig. S11(A-D)). Results show that the network predicted from randomized features had fewer TFs with a significant GO term, and much lower performance than the original NP3 network with the integration method using the GO, GO-directness and PPI evaluation metrics. These results suggest that by just overfitting the binding labels, we will not be able to produce improved performance using our evaluation metrics. Features are from DE and regression data and hence they are enriched with functional edges, which probably what helped XG-boost in finding a consensus between this data and the binding data. The predicted TF network map using the integration mode has a better performance and more enriched with direct and functional edges.

References

Bischl, B., et al. mlr: Machine Learning in R. The Journal of Machine Learning Research 2016;17(1):5938-5942.
Chen, T. and Guestrin, C. Xgboost: A scalable tree boosting system. In, Proceedings of the 22nd acm sigkdd international conference on knowledge discovery and data mining. 2016. p. 785-794.
Hackett, S.R., et al. Learning causal networks using inducible transcription factors and transcriptome-wide time series. Mol Syst Biol 2020;16(3):e9174.
Kang, Y., et al. NetProphet 2.0: Mapping Transcription Factor Networks by Exploiting Scalable Data Resources. Bioinformatics 2017.
Kemmeren, P., et al. Large-scale genetic perturbations reveal regulatory networks and an abundance of gene-specific repressors. Cell 2014;157(3):740-752.
